# Associating a product with a luxury brand label modulates neural reward processing and favors choices in materialistic individuals

**DOI:** 10.1038/s41598-017-16544-6

**Published:** 2017-11-23

**Authors:** Catherine Audrin, Leonardo Ceravolo, Julien Chanal, Tobias Brosch, David Sander

**Affiliations:** 1 Swiss Center for Affective Sciences, Campus Biotech, Geneva, Switzerland; 2 Laboratory for the Study of Emotion Elicitation and Expression (E3 Lab), Department of Psychology, University of Geneva, Geneva, Switzerland; 3 Methodology and Data Analysis group, Department of Psychology, University of Geneva, Geneva, Switzerland; 4 Neuroscience of Emotion and Affective Dynamics laboratory, Department of Psychology, University of Geneva, Geneva, Switzerland; 5 Consumer Decision and Sustainable Behavior Laboratory, Department of Psychology, University of Geneva, Geneva, Switzerland; 6 University of Teacher Education, Lausanne, Switzerland

## Abstract

The present study investigated the extent to which luxury vs. non-luxury brand labels (i.e., extrinsic cues) randomly assigned to items and preferences for these items impact choice, and how this impact may be moderated by materialistic tendencies (i.e., individual characteristics). The main objective was to investigate the neural correlates of abovementioned effects using functional magnetic resonance imaging. Behavioural results showed that the more materialistic people are, the more they choose and like items labelled with luxury brands. Neuroimaging results revealed the implication of a neural network including the dorsolateral and ventromedial prefrontal cortex and the orbitofrontal cortex that was modulated by the brand label and also by the participants’ preference. Most importantly, items with randomly assigned luxurious brand labels were preferentially chosen by participants and triggered enhanced signal in the caudate nucleus. This effect increased linearly with materialistic tendencies. Our results highlight the impact of brand-item association, although random in our study, and materialism on preference, relying on subparts of the brain valuation system for the integration of extrinsic cues, preferences and individual characteristics.

## Introduction

Research on consumer preferences has revealed the importance of extrinsic cues when evaluating the quality of an item^1^. Extrinsic cues refer to any piece of information about the item that is not directly part of the item itself^2^, such as its price or the label displayed on it. When evaluating strictly identical items displayed with different prices, consumers prefer high-priced items^3^. Similarly, items with a green label are preferred over items that are intrinsically identical, but presented with a regular label: consumers choose them more often, accept to pay more for them, and – in the case of food items - report them to taste better^4–8^. Other studies showed that consumers’ expectancies associated with a brand impacted experienced pleasantness when consuming the item^9,10^. Specifically, when participants drank Coke and Pepsi without knowing which one they were drinking, their experienced pleasantness was equal for both drinks. However, when drinks were labelled with their brand, participants reported increased preference for Coke over Pepsi^10^. Thus, labels and brand information, by assigning a value to an item, can have a large impact on consumers’ preferences.

Preference is a subjective value that refers to the worthiness and pleasantness associated with the stimulus that is evaluated and, with respect to behaviour, typically leads to the choice of one option over another^4,11^. The neural network underlying the computation of subjective values has been described in numerous functional magnetic resonance imaging (fMRI) studies. Key regions of this “brain valuation system” are the ventral striatum (VS), ventromedial prefrontal cortex (VMPFC), orbitofrontal cortex (OFC), insula, amygdala, and the posterior cingulate cortex^12–17^. Crucially, in their meta-analysis, Bartra *et al*. revealed that subjective values were reliably correlated with neural activity in the VS and VMPFC^12^. The aforementioned regions are part of a network that computes appetitive and aversive values^18^ as well as primary and secondary rewards^19^ such as food or money^19^, cars^20^, faces^21^, and social cues (e.g. one’s reputation^22^). Previous experiments have revealed that activity in the brain valuation system can be modified by information that is extrinsic to the stimulus. For instance, tasting wine presented with a higher price tag increased medial OFC and VMPFC activity^3^; displaying the brand of an item modified dorsolateral prefrontal cortex (DLPFC) and hippocampus activity^10^; displaying an organic label increased activity in the striatum and in the DLPFC^5^. However, Kirk *et al*.^23^ demonstrated that when participants were viewing works of art, the neural impact of extrinsic information was dependent on the participant’s expertise in art. Specifically, extrinsic information increased activity in the DLPFC in experts and VMPFC in non-experts. Therefore, activity in the brain valuation system has been shown to be dependent on both extrinsic information about the stimuli and individual characteristics^3,5,10,23^. When it comes to choices and purchase decisions, previous studies suggest that individual characteristics of the consumer may lead to increased interest and attention toward brands^24^. More specifically, materialism is positively related to brand connection^24^, brand dependence^25^ and leads to higher consideration of luxury brands^26^.

In the present study, we specifically aimed at investigating a “luxury brand effect”, namely the extent to which certain types of brand labels (luxurious/non-luxurious) displayed with items may have an impact on the brain valuation system and on choices. We further wanted to test whether this process may be modulated by materialism. Participants were presented with items displayed either with a luxurious or with a non-luxurious brand label and were asked to assess how much they liked them while lying in a magnetic resonance imaging scanner. In an independent offline task, participants were asked to choose between items displayed either with a luxurious or with a non-luxurious brand label. Critically, items were *randomly* associated with either a luxurious or a non-luxurious brand across participants in order to avoid any association between a specific object and a brand. In line with the abovementioned body of work revealing the impact of extrinsic cues on preference, we hypothesized that brands would have an impact on preferences. More specifically, we hypothesized that items displayed with a luxurious brand, as opposed to a non-luxurious brand label, would be evaluated more positively and chosen more often. We additionally hypothesized that, the more materialistic participants are, the more often they would like and choose items displayed with luxurious brands. Regarding neuroimaging results, we aimed at testing whether brand labels (luxurious vs non-luxurious) have a differential impact on brain activity depending on the liking ratings (preference; Most vs Least liked). More specifically, we predicted that the evaluation (Most vs Least liked) of luxury brands as compared to non-luxury brands would trigger enhanced activity in the brain valuation system, namely in the VS and VMPFC. Finally, we predicted that materialism would enhance activity in the striatum (ventral and/or dorsal) and VMPFC when choosing items displayed with a luxurious as opposed to a non-luxurious brand label.

## Results

### Behavioural results: Liking task

Analyses revealed a significant main effect of the brand label (b = 25.794, CI95% = [10.547; 41.054], t(37.890) = 3.309, p = 0.002), suggesting that items displayed with luxury brand labels were preferred to those displayed with non-luxury brands, although the items were exactly the same across conditions with brand labels randomly assigned. Concerning our second hypothesis, results revealed a significant interaction between materialism and brand label (b = 14.437, CI95% = [1.251; 27.615], t(35.410) = 2.144, p = 0.039), suggesting that the impact of the brand label increased with materialism. In other words, the more materialistic the participants, the more they liked items displayed with a luxurious over a non-luxurious brand.

### Behavioural results: Forced-choice task

**Figure 1:**
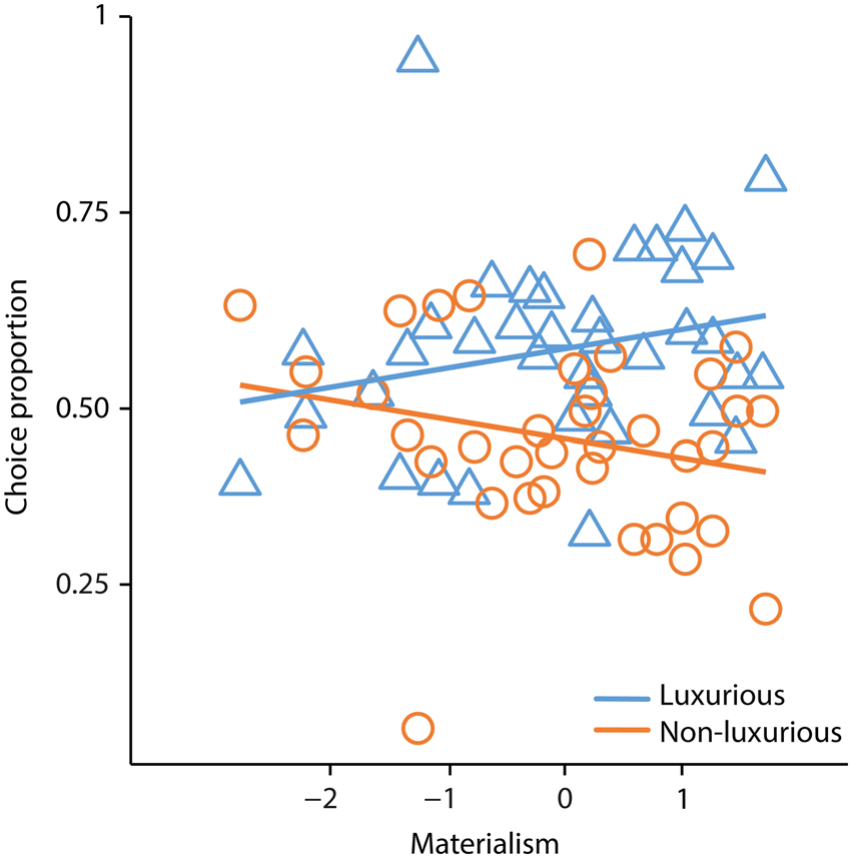
Behavioural results illustrating the proportion of choice (Y axis) as a function of materialism (X axis) for luxurious (blue triangles) vs. non-luxurious brands (orange circles). Lines represent the fitted regression line for luxurious (blue line) and non-luxurious brands (orange line).

As reported in Table 1, analyses revealed a significant main effect of the brand label (b = 0.299, CI95% = [0.184; 0.415], z = 5.094, p < 0.001), revealing that luxurious brands were chosen more frequently than non-luxurious brands. Moreover, analyses revealed a main effect of liking (b = 0.629, CI95% = [0.517; 0.742], z = 10.980, p < 0.001), showing that most liked objects were more often chosen than least liked objects. Results revealed a significant interaction between materialism and brand (b = 0.117, CI95% = [0.015; 0.221], z = 2.245, p < 0.0248), suggesting that the higher the materialistic tendencies, the more participants choose items presented with luxurious brands more often than items presented with non-luxurious brands (see Fig. 1). No further significant effects were found (all p > 0.05).

**Table 1:** Behavioural results, main effects and interactions.

*Fixed Effects*	*b*	*SE*	*p-value*
*Intercept*	*−0*.*008*	*0*.*069*	*0*.*908*
*Brand*	*0*.*299*	*0*.*059*	*0*.001***
*Liking*	*0*.*629*	*0*.*057*	*0*.001***
*Materialism*	*0*.*007*	*0*.*050*	*0*.*879*
*Brand* × *Materialism*	*0*.*147*	*0*.*051*	*0*.004**
*Brand* × *Liking*	*0*.*005*	*0*.*567*	*0*.*928*
*Liking* × *Materialism*	*0*.*036*	*0*.*050*	*0*.*468*
*Brand* × *Liking* × *Materialism*	*0*.*032*	*0*.*05*	*0*.*526*
***Random Effects***	***σ2***	***SE***	
*Participants*			
*Intercept*	*0*.*001*	*0*.*001*	
*Images on the left*			
*Intercept*	*0*.*113*	*0*.*4336*	

Summary of mixed-effects model analyses for predicting item choices as a function of Liking, brand and materialism. ***p < 0.001; **p < 0.01.

### Imaging results

#### Model 1: Liking and Brand label factors

**Figure 2:**
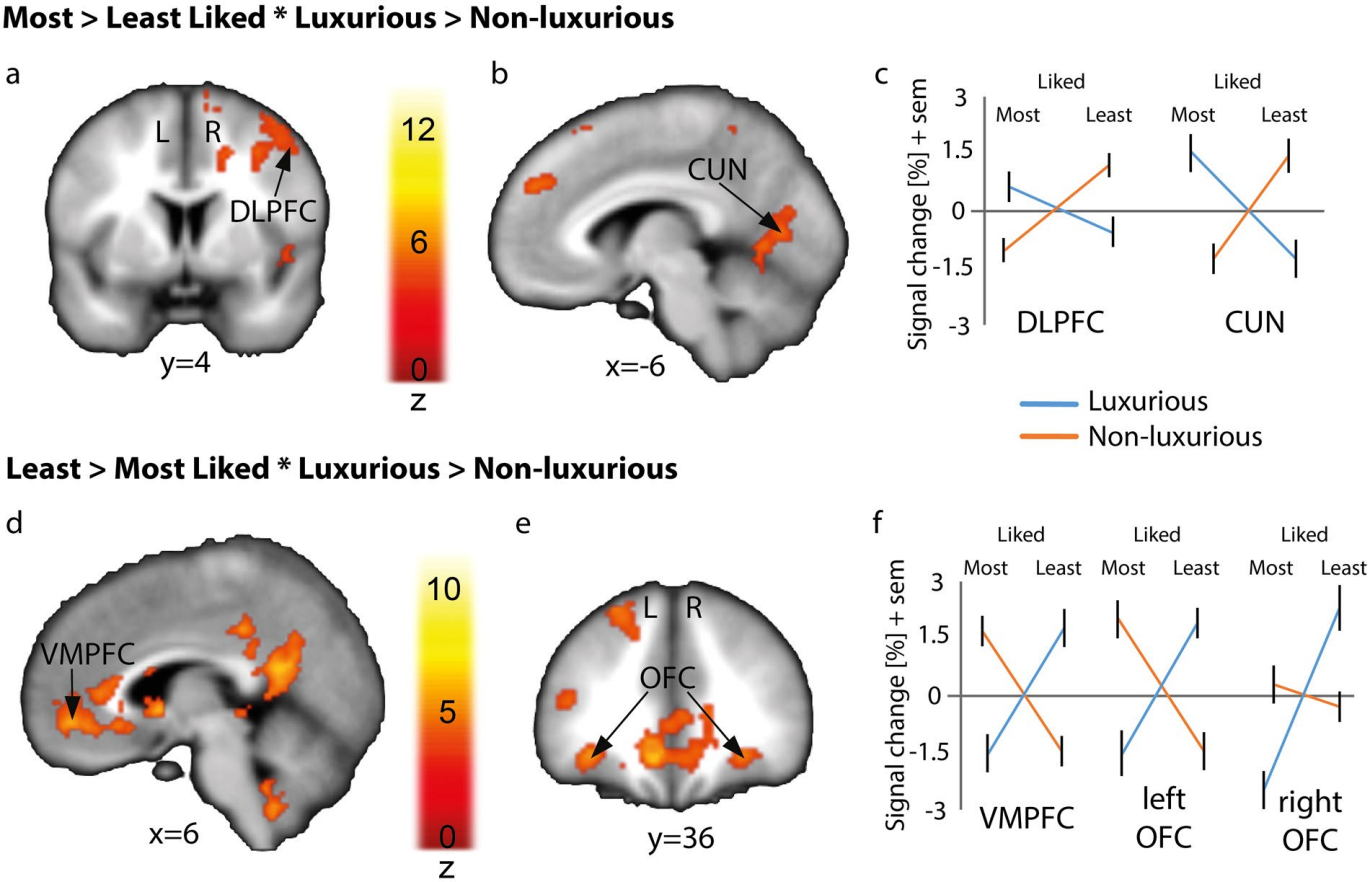
Interaction between “Liking” and “Brand label” factors in the brain. Enhanced BOLD signal for Most liked > Least liked * Luxurious > Non-luxurious items (see Table 2) in the dorsolateral prefrontal cortex (**a)**; DLPFC) and cuneus (**b**). For the inverse contrast (Least liked > Most liked * Luxurious > Non-luxurious; see Table 3), activations were observed in the ventromedial prefrontal cortex (**c)**; VMPFC) and bilaterally in the orbitofrontal cortex (**d)**; OFC). Percentage of signal change for the DLPFC and Cuneus (**c**), VMPFC and bilateral OFC (**f**), in which error bars represent one standard error of the mean (sem) for luxurious (blue line) and non-luxurious (orange line) items. Whole brain activations are displayed at p < 0.05 (voxel-wise FDR) with a cluster extent of k > 50. The colored bars represent the statistical Z value of the contrast. CUN: cuneus.

The interaction between Liking and Brand label (Fig. 2) was used to highlight enhanced signal for luxurious compared to non-luxurious items when these were most liked as opposed to when they were least liked. We hence contrasted Most liked > Least liked * Luxurious > Non-luxurious items and observed enhanced BOLD (Blood-Oxygenation Level-Dependent signal) signal in the postcentral gyrus, dorsolateral prefrontal cortex (DLPFC, Fig. 2a,c), ventrolateral prefrontal cortex (VLPFC), cuneus (Fig. 2b,c) and anterior cingulate cortex (ACC; See Table 2 for all regions). Results specifically in the DLPFC and cuneus were also found for Most liked > Least liked for Luxurious items (DLPFC: MNI xyz 34 50 16, z = 5.92; Cuneus: MNI xyz 12–68 14, z = 5.25; Contrast thresholded at p < 0.05 FDR corrected, k > 50). The inverse contrast (Least liked > Most liked * Luxurious > Non-luxurious) yielded enhanced signal in the bilateral parahippocampal gyrus, ventromedial prefrontal cortex (VMPFC; Fig. 2d,f), bilateral lateral OFC (Fig. 2e,f) and posterior cingulate cortex (Fig. 2d; See Table 3 for all regions). Among these regions, the posterior cingulate cortex (PCC), the VMPFC and the bilateral OFC were also found specifically for Least liked > Most liked for Luxurious items in addition to the bilateral amygdala and the left hippocampus (PCC: MNI xyz -6–56 14, z = Inf.; VMPFC: MNI xyz 10 46 -8, z = 6.99; OFC: MNI xyz -32 36 -10 and 28 36 -10, z = 6.48 and z = 6.69, respectively; Amygdala: MNI xyz -22 -18 -16 and 22 -16 -22, z = 6.19 and z = 7.01, respectively; Hippocampus: MNI xyz -30 -40 -10, z = 7.38; Contrast thresholded at p < 0.05 FDR corrected, k > 50).

**Table 2:** Interaction Effect for Most liked > Least liked * Luxurious > Non-luxurious.

Region Name	Side	MNI			FDR p < 0.05, k = 50	
		X	Y	Z	Z value	Number of voxels
Postcentral Gyrus	L	−46	−18	50	Inf	595
Superior Temporal Gyrus	R	54	−40	22	6.40	1028
Precuneus	R	10	−58	62	6.12	495
Insula	R	30	−26	20	5.47	64
Cuneus	L	−6	−74	8	5.37	566
Cingulate Gyrus	L	−14	16	28	5.36	36
Anterior Cingulate	L	−6	50	30	5.33	90
Orbitofrontal cortex	L	−32	24	−11	5.25	59
Cingulate Gyrus	R	22	6	44	5.11	73
Supramarginal Gyrus	L	−52	−50	22	5.05	180
Inferior Frontal Gyrus	R	50	22	12	5.01	141
Superior Frontal Gyrus	L	−10	20	62	5.00	90
Superior Frontal Gyrus	R	36	52	18	4.86	544
Middle Frontal Gyrus	R	46	56	10	4.77	96
Insula	R	32	−6	26	4.70	70
Insula	R	34	12	10	4.58	52
Inferior Frontal Gyrus	R	56	40	0	4.38	58
Insula	L	−44	10	−2	3.93	52

Mean cluster location and local maxima of BOLD signal change for liked compared with disliked in interaction with luxurious compared to non-luxurious condition in the fMRI Liking task (p < 0.05, FDR corrected, k > 50).

**Table 3:** Interaction Effect for Least liked > Most liked * Luxurious > Non-luxurious.

Region Name	Side	MNI			FDR, p < 0.05, k = 50	
		X	Y	Z	Z value	Number of voxels
Posterior Cingulate	L	−6	−54	16	Inf	2240
Anterior cingulate	L	−8	−42	6		
Postcentral Gyrus	L	−38	−26	60	7.36	352
VMPFC	L	−10	36	−10	7.10	2362
Caudate	L	−8	10	−2	6.48	
Superior Frontal Gyrus	L	−22	40	48	6.07	654
Precuneus	L	−32	−72	40	5.88	678
Middle Temporal Gyrus	L	−64	−2	−18	5.70	419
Precentral Gyrus	R	36	−22	62	5.37	145
Superior Frontal Gyrus	R	20	−12	66		
Putamen	L	−24	0	2	5.34	165
Caudate	R	10	14	2	5.27	
Middle Frontal Gyrus	R	28	34	−10	5.18	150
Parahippocampal Gyrus	R	28	−30	−18	5.10	318
Insula	L	−40	−4	4	5.08	78
Cuneus	L	−10	−94	4	5.01	125
Middle Frontal Gyrus	L	−36	32	20	4.78	110
Inferior Temporal Gyrus	R	60	−8	−22	4.50	128
Middle Temporal Gyrus	R	40	−68	22	4.10	130

Mean cluster location and local maxima of BOLD signal change for disliked compared with liked in interaction with luxurious compared to non-luxurious condition in the fMRI Liking task (p < 0.05, FDR corrected, k > 50).

#### Model 2: ROI analysis in the caudate nucleus of Liking and Brand label factors

**Figure 3:**
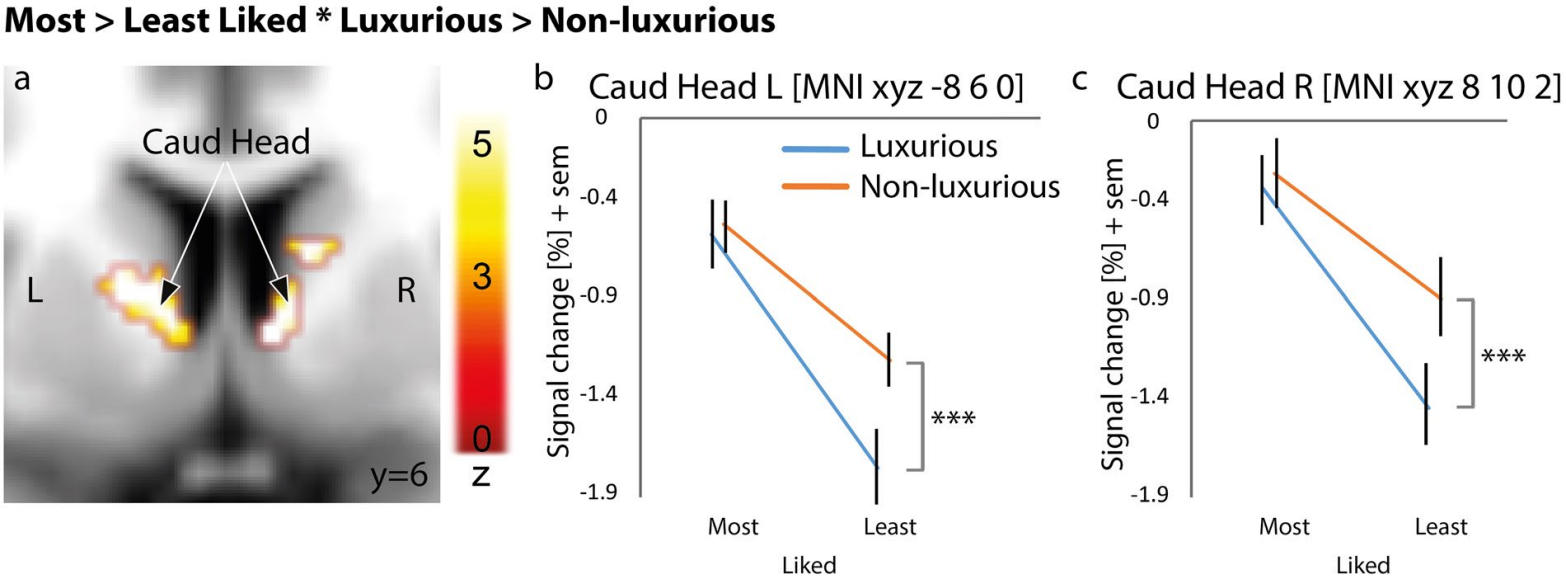
Localization of value-related areas in our main liking task using a mask (region of interest analysis) obtained through a functional localizer task, namely the Monetary Incentive Delay task. Region of interest analysis in the left and right head of the caudate thresholded at p < 0.05 Family-Wise Error corrected at the voxel level (**a**). Extracted percentage of signal change for the contrast Most liked > Least liked * Luxurious > Non-luxurious in the left (**b**) and right caudate head (**c**). Blue lines represent luxurious items, non-luxurious items are illustrated by the orange line. Error bars represent one standard error of the mean (sem). The colored bars represent the statistical Z value of the contrast. Caud Head: Caudate head. MNI: Montreal Neurological Institute. ***p < 0.001.

In this model, we computed ROI analyses based on the functional Monetary Incentive Delay (MID) task^27^ localizer in order to investigate the impact of Liking and Luxury factors in our sample-specific reward-related brain regions, namely the bilateral caudate head regions. Activity in the left and right caudate (MNI xyz -8 6 0 and 8 10 2, respectively) revealed in both cases a significant effect of Liking (Fig. 3a), where most liked products were related to less deactivation than least liked products (*F*(1, 36) = 18.095, *p* < 0.001; *F*(1, 36) = 20.191, *p* < 0.001). In both regions, the difference between most liked and least liked product was more important for items with a luxurious label (mean difference = −1.186, p < 0.001; mean difference = −1.119, *p* = 0.003) than for items labelled as non-luxurious (mean difference = −0.536, *p* = 0.08; mean difference = −0.636, *p* = 0.154; See Fig. 3b,c).

#### Model 3: Brand label, Choice and Materialism

**Figure 4:**
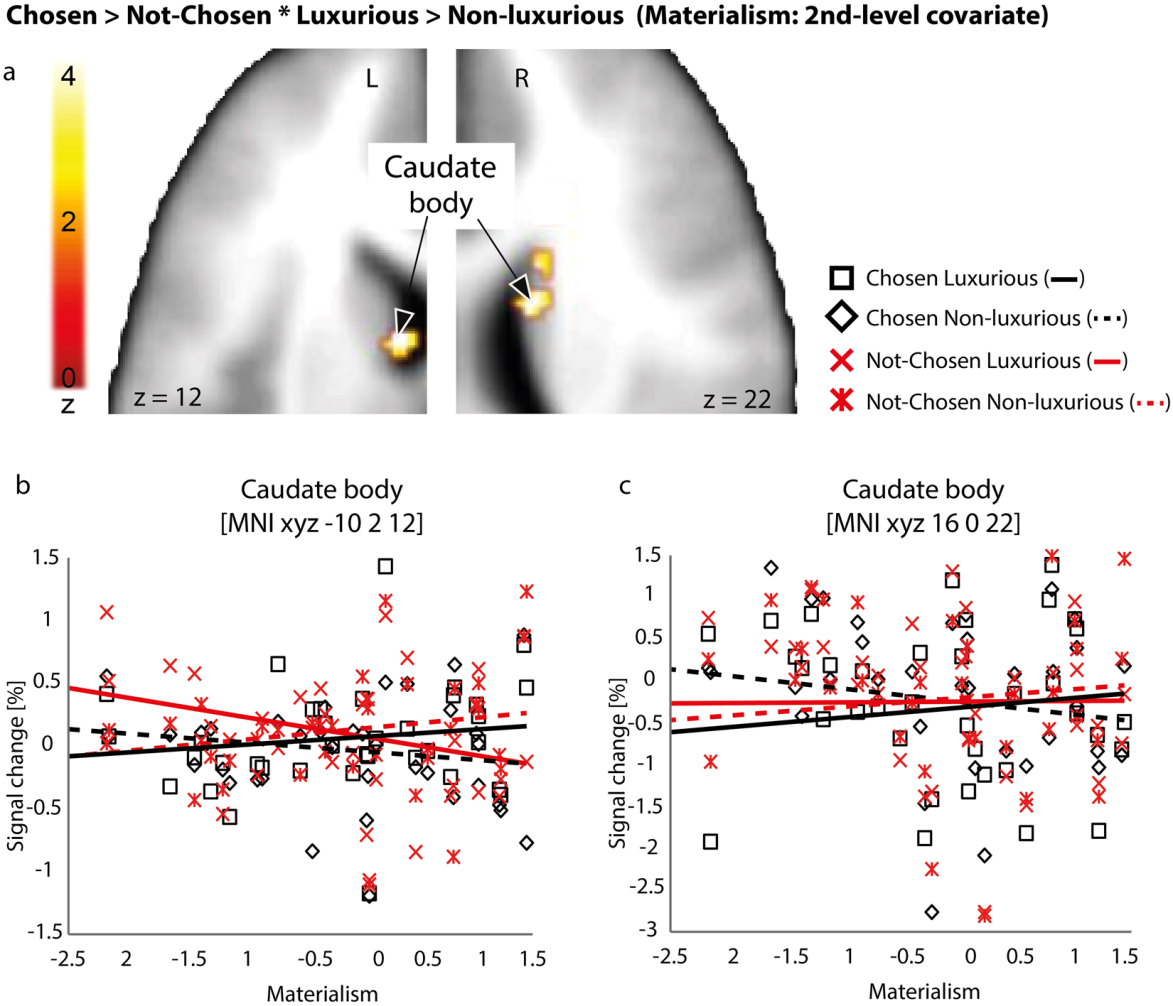
Interaction between Chosen > Not-chosen * Luxurious > Non-luxurious using materialistic tendencies’ scores for each participant as a second-level covariate in the bilateral caudate body (**a**), thresholded at Psvc < 0.05 (voxel-wise FDR) using a mask including the ventral, dorsal striatum and the ventromedial prefrontal cortex. Percentage of signal change in the left (**b**) and right (**c**) caudate body. To illustrate the effect of materialism, panels (b,c) include a scatter plot in which each point represents, for each condition, the mean value of percentage of signal change for each individual participant (Y axis) according to his/her materialism score (X axis): a negative value indicates very low materialistic tendencies; a positive value indicates high materialistic tendencies; ‘0’ corresponds to an average level of materialism. Lines represent a linear regression tendency computed for each condition separately. Black shapes represent items chosen preferentially in the offline choice task (Squares/solid line: Luxurious items; Tilted squares/dashed line: Non-luxurious items). Red crosses represent items that were not chosen in the choice task (Normal red cross/solid line: Luxurious items; Modified red cross/dashed line: Non-luxurious items). The colored bar represents the statistical Z value of the contrast. MNI: Montreal Neurological Institute.

Model 3 focused on the differences in cerebral activation depending on whether the item was later chosen, while considering brand label and participants’ materialism levels. Materialism was used as a second-level covariate in order to gain power through participant scores (More vs Less materialistic based on a continuum rather than based on artificially created groups). Model 3 revealed a significant interaction (Chosen > Not-chosen * Luxurious > Non-luxurious; Fig. 4) in the left (MNI xyz -10 2 12, z = 3.72, Psvc = 0.045 FDR-corrected) and right (MNI xyz 16 0 22, z = 4.08, Psvc = 0.045 FDR-corrected) caudate body (Fig. 4a). Results revealed that the abovementioned 2-way interaction varied as a function of Materialism in both left (Fig. 4b) and right (Fig. 4c) caudate body. More specifically, the more participants were materialistic, the more enhanced the activity in the caudate nucleus for items displayed with a luxury brand that were later chosen.

## Discussion

In the present study, we aimed at investigating the extent to which extrinsic cues (i.e., brand labels), preference (i.e., item liking) and individual characteristics (i.e., materialism) may interact to further impact on activity patterns of the so-called “brain valuation system”^12^. Crucially, our manipulation consisted in the mere randomized association of items with either luxurious or non-luxurious brand labels across participants. Behavioural results showed an impact of the brand label manipulation, which was modulated by participants’ materialism, on preference and choice. Neuroimaging data revealed a neural modulation of the brain valuation system by the participants’ preference (i.e., item liking) together with the brand manipulation, most notably in the DLPFC, cuneus and bilateral caudate head. Further analyses revealed that the more participants were materialistic, the more enhanced the activity in the caudate nucleus for luxury-labelled items that were later chosen.

Behavioural results revealed that, on average, participants preferred, and chose more often, items displayed with luxurious brand labels, although the items were physically identical across conditions (luxurious and non-luxurious brand labels were randomly assigned to the presented items across participants). Hence, this first result provides evidence for our hypothesized “luxury brand effect” and highlights the importance of the label associated with an item or event: the mere and random association with a luxury brand label was sufficient to influence the participants’ preference. Moreover, our results revealed that materialistic tendencies could modulate the importance of brand labels during preference evaluation and choices, as the impact of brand labels increased with materialism. These results provide a replication of previous evidence for such an effect^26^, while extending them to a forced-choice task. Taken together, these results add further weight for the importance of extrinsic cues when making decisions^3,10^. Importantly, our behavioural results revealed that sensitivity toward extrinsic cues also depends on individual characteristics, as previously pointed out in the literature^26,28^.

Neuroimaging results highlighted the importance of both preference and brand labels (which varied randomly across participants and stimuli in the present study). This experimental manipulation impacted on cerebral regions involved in visual attention (i.e., the cuneus^29^) for most liked items displayed with a luxurious label, suggesting a potential facilitated attentional toward such items. Interestingly, a similar pattern of activation was observed in the anterior part of the cingulate cortex (ACC), which was previously shown to underlie relevant stimulus attribution^30^. Our manipulation also yielded enhanced activity in regions linked to subjective value assessment^31^ and reward processing (i.e., the caudate head^32^; the cuneus^33^; the orbitofrontal cortex^34^; the VMPFC^10^ and the amygdala^16,34^). Interestingly, our results further highlight the implication of the DLPFC for most liked stimuli displayed with a luxurious brand. This result echoes the findings of McClure *et al*.^10^ who pointed out that brand knowledge had an impact on activation in the DLPFC. More generally, this region may also be recruited in cognitive control of affective states^35^.

The interaction between liking and luxury observed in the VMPFC is in accordance with previous evidence highlighting an interaction between sensory processes and top-down information in this region^36^. Our results are thus in line with previous accounts showing the importance of the VMPFC as a hub, integrating affective and conceptual information^37^. In our case, affective information may refer to the evaluation of the presented item associated with a brand, while conceptual information may refer to the processing of the luxury dimension of the brand (and to the sense of quality and social status it implies). Our results also pointed out the implication of the hippocampus. This region, crucially related to memory, is involved in contextual information integration^10^, valuation of imagined outcomes^38^ and more generally in value-based decision-making^39^, this latter result being in-line with our study design and findings. Finally, activation in the PCC was highlighted as well. The role of this structure has received less attention for encoding value, but studies suggest that the PCC is functionally coupled with the VMPFC, even though these regions may have independent functions in valuation computation^30^. This interpretation should however be tested and future studies should investigate the specific importance of PCC in value-based decision-making.

More generally, our results for model 1 and 2 (interaction between liking and luxury) emphasize the role of the DLPFC and the hippocampus in valuation, which is potentially related to the necessity of integrating brand knowledge for preference computation. Crucially, our results point out that information about the brand is also integrated in the caudate nucleus. While this region is known to be strongly linked to preference ratings (i.e., liking^40^), we show that it also integrates information such as brand knowledge. This suggests that, despite evolutionary ancient, the caudate nucleus integrates modern cultural cues (brands), echoing results from previous research suggesting that activity in the mesolimbic reward system is modulated by culture^41,42^.

Moreover, our last model revealed that the more materialistic participants were, the more active the bilateral caudate nucleus was (caudate body) for luxurious compared to non-luxurious items that would later be chosen (as opposed to not-chosen). This result suggests that more materialistic participants recruit reward-related areas when acting congruently with their materialistic values (i.e., when they choose items associated with luxury brand labels, as materialistic people tend to value luxury^43^). To summarize, our data provide evidence for the importance of individual characteristics in modulating the activation and involvement of the “brain valuation system”^44^, in addition to the impact of the context (i.e., item labelled as luxurious vs. non luxurious in our study) and the individual’s behavioural preference (i.e., choosing an item or discarding it).

While the abovementioned data address our hypotheses, our results still reveal some discrepancies between neuroimaging and behavioral results. In fact, although we showed that the impact of liking was modulated by the brand label in the fMRI results, we observed no interaction between brand and liking in the behavioral results regarding choice. Interestingly, Sharot *et al*.^45^ also faced a difference between BOLD signal and overt measures of preferences. In their experiment, they observed differences in the BOLD signal associated with a choice, but no such result was observed behaviorally. As they suggest, “the very fact that a preference was not expressed behaviorally […] does not mean that it was not experienced” (Sharot *et al*.^45^, p. 3764). Future studies may specifically test how neural activation observed in the situation of automatic evaluation may predict future choice or decisions, for instance on a trial-by-trial basis^35^. Other limitations of our study have to be considered as well. First, we studied the impact of brands, thus focusing our interest and experimental manipulation on one single extrinsic cue. While this was necessary to address our “luxury brand effect” hypothesis, preference and choices are usually made based on more than one extrinsic aspect. People usually take into account other extrinsic cues (e.g., price, expert ratings, information about the country of origin). Thus, future studies should integrate and assess the importance of several extrinsic cues on preference and on the activation of the brain valuation system. Second, product pictures and brand labels were presented together and at the same time to the participants, thus making it difficult to dissociate the processing of intrinsic cues (i.e., product-specific preference) from the processing of extrinsic cues (i.e., the brand). An interesting future study could first present the product, later followed by the brand. This may help to assess measurable differences in preference based first on intrinsic and then on extrinsic cues. Finally, our sample was exclusively constituted of female students. While it was motivated by the products we chose (i.e., exclusively designed for women), future studies should evaluate how such results may apply to male and/or other participants who would be more familiar with luxury consumption.

The purpose of the present study was to assess how individual preferences (i.e., item liking and choice), extrinsic cues (i.e., brand labels) and personal values (i.e., materialism) could modulate activity in the “brain valuation system”. Our results highlight the extent to which extrinsic cues such as brand labels can, even when randomly assigned to certain items, modulate preferences at the behavioural and brain levels. In particular, brand processing and individual preferences were found to interact in regions typically involved in subjective value processing such as the DLPFC, the caudate head, the OFC, and the VMPFC. Crucially, our results suggest an important role played by the bilateral caudate nucleus for integrating the luxury dimension associated with the brand, the individual preferences, and the personal materialistic values.

## Methods

### Participants and procedure

Participants were recruited among students of the University of Geneva. Thirty-eight healthy right-handed female participants (mean age = 23.33, range = 18–32) with no history of psychological or neurological disorders were selected from a larger sample. All 38 participants performed the liking task and monetary incentive delay task (see below), but only 37 were included in the offline choice task due to a corrupted logfile that was unreadable. Because of the nature of our stimuli (i.e., items strictly designed to be worn by women), only female participants were recruited. The study was performed according to the rules and regulations of the University of Geneva and the declaration of Helsinki, and through official approval by the Cantonal Ethics Committee. All participants gave their written informed consent to take part in the study after careful examination of details regarding the study. They were also informed they had no obligation to finish the session and were free to leave at any time they wished, was the situation uncomfortable to them.

**Figure 5:**
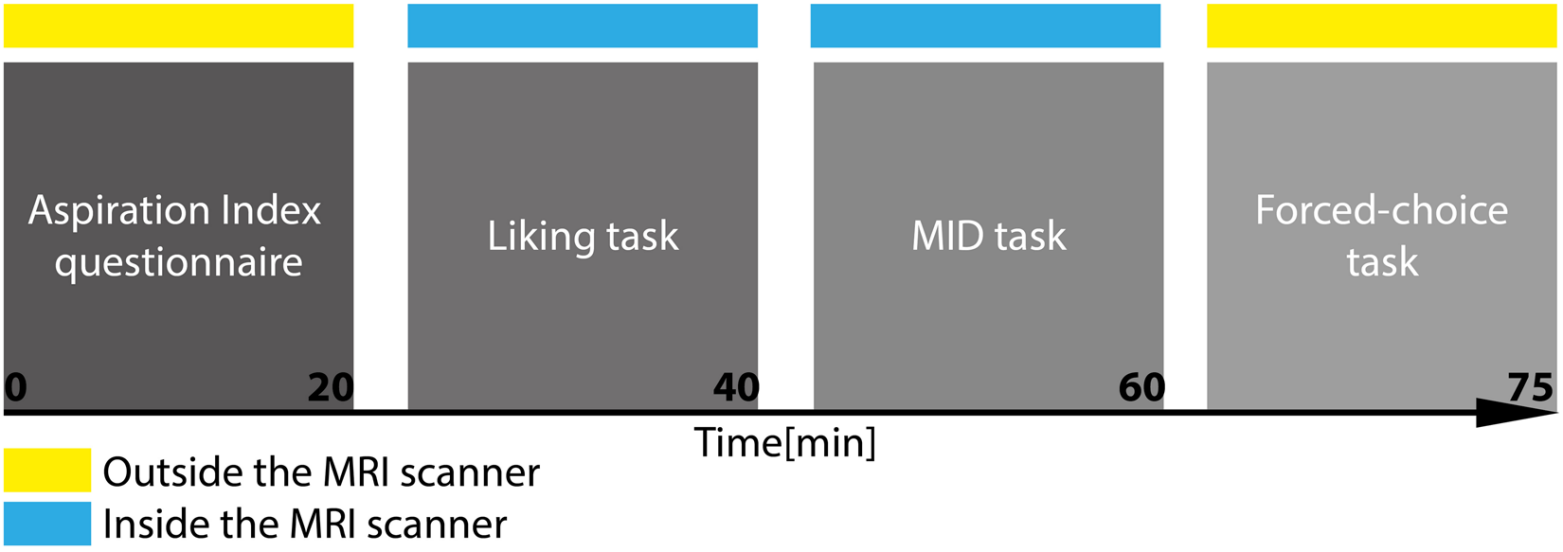
Experimental timeline for a complete session in minutes (X axis) for one participant, showing the order of task presentation and specifying whether the task was performed inside (blue rectangles) or outside the MRI scanner (yellow rectangles). MID: Monetary Incentive Delay task.

Participants completed an online questionnaire containing the Aspiration Index as well as several demographic questions about their age and income (all participants had similar income, i.e. annual income below 10000US$). When coming to the lab, participants first were informed about the procedure. Once in the scanner, they performed a liking task followed by a functional localizer task (the Monetary Incentive Delay task^27^) and a forced-choice task outside the scanner (see Fig. 5 for the experimental timeline).

## Materials

### Materialism

Participants completed the Aspiration Index questionnaire^46,47^, a questionnaire designed to measure materialism, which is highly correlated with other measures of materialism^48^. They were asked to assess how important 17 goals are to them on a scale ranging from 1 (“not important at all”) to 9 (“extremely important”). These goals refer to 4 dimensions such as the importance that one gives to one’s image (e.g. “I hope for the future that my image will be one that others find appealing”), popularity (e.g. “I will be admired by many people”), financial success (e.g. “I will have expensive possessions”), and conformism (e.g. “I will live up to the expectations of my society”). Participants’ scores of materialism was computed by averaging their scores on each dimension^46,48^.

### Pre-test study

We performed a pre-test study in order to select the items presented to the participants during the liking task in the scanner. The purpose of this pilot study was to select images of items having a similar level of perceived quality. Thus, we selected the stimuli presented in Experiment 1 in Audrin *et al*.^26^, to which we added new stimuli to reach 120 items of ready-to-wear items (i.e., scarves, handbags, belts and purses) and presented them in an online experiment. Participants had similar characteristics to the sample of participants recruited for the fMRI study (i.e. women with the same age and demographic characteristics). Participants were presented with one item at a time and requested to assess how high they perceived the quality of the item, based on the picture only. The scale was ranging from −100 (“very low quality) to 100 (“very high quality”). We selected the 80 items with the highest quality evaluation (median = 11.750, sd = 14.5). This pilot study ensured us that the selected stimuli had a similar level of perceived quality.

### Experimental procedure: fMRI liking task

**Figure 6:**
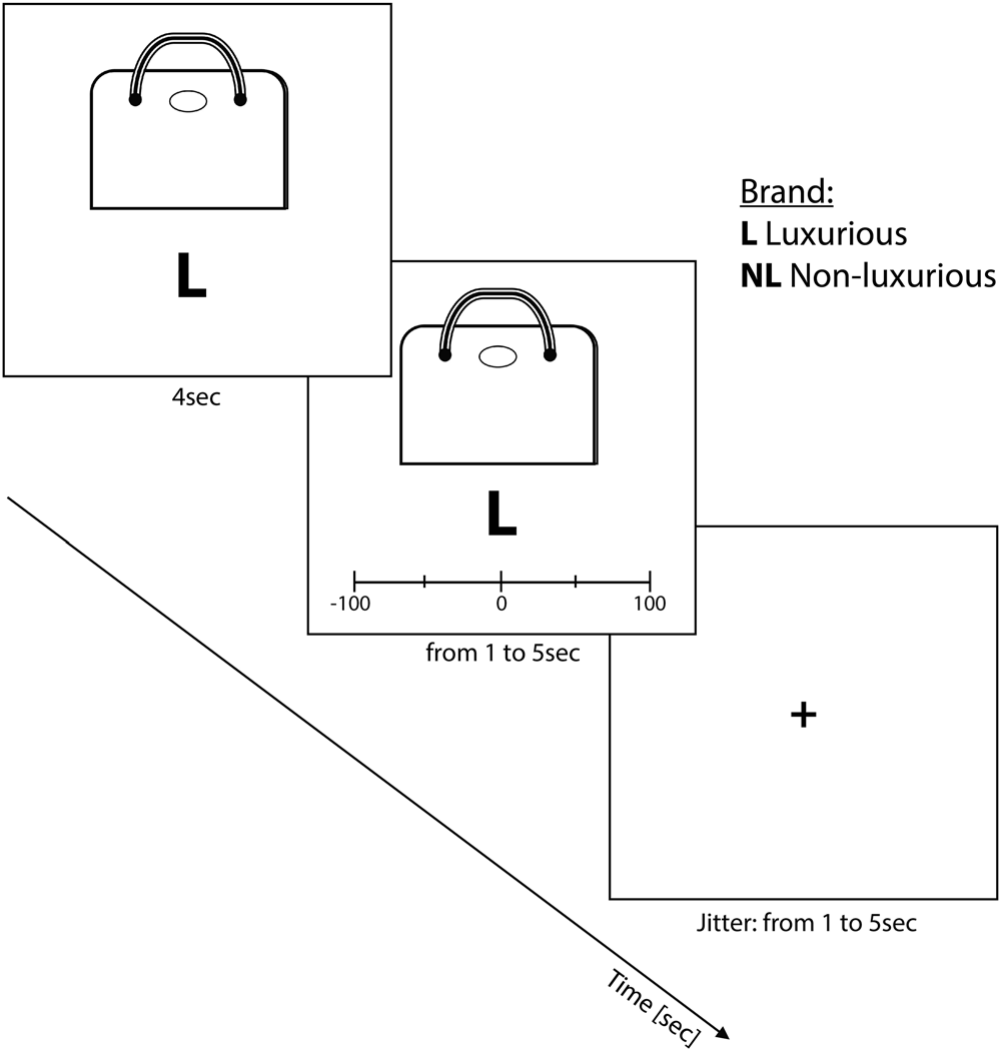
Description of the experimental task. Participants first saw the item with its brand during 4 seconds; then they were asked to assess how much they liked the item by moving the slider from −100 (I really don’t like it) to +100 (I really like it). They could answer during maximum 5 seconds. Then, a fixation cross was displayed during 1 to 5 seconds, so that the time between the evaluation and the next trial lasted maximum 10 seconds. Neuroimaging volumes were acquired continuously during the task.

For the liking task, participants were lying in the scanner and images of ready-to-wear items (i.e., scarves, handbags, belts and purses) were presented on a screen one after the other (See Fig. 6 for details). Each item was presented with 1 out of 8 brand labels: 4 luxurious and 4 non-luxurious^26^. All the brands principally manufactured clothing. Their luxurious vs. non-luxurious categorization was based on rankings such as the GenY Prestige Brand Ranking^49^, which ranks the top luxurious clothing brands for women. Thus, brands appearing in this ranking were categorized as luxurious whereas brands which did not appear in this ranking were categorized as non-luxurious. The luxurious and non-luxurious brands were pseudo-randomized between participants so that each item was seen once with each of the brands. The task consisted in 80 trials (stimuli selected following the pre-test study), 40 of which were presented in the luxurious condition while 40 others were presented in the non-luxurious condition. Each trial started by a fixation cross. Afterwards, an item and a brand were displayed on the screen during 4 seconds. After these 4 seconds, a scale was displayed below the item and participants were asked to rate the item by moving the slider either toward the “I don’t like it” end, or toward the “I really like it” end. Once participants had made their evaluation, they clicked on a button to start the next trial. A fixation cross appeared and the time of presentation of this cross was adjusted with the time participants spent giving their answer so that each trial lasted 10 seconds. Responses were made using a four button MR-compatible response box (Current Designs Inc., Philadelphia, PA, USA), where the first button allowed participants to move the slider toward the left, the last button allowed them to move the slider toward the right and the two buttons in the middle allowed them to confirm that they had rated the item and that they were ready to rate the following item.

### Experimental procedure: fMRI Money Incentive Delay task

After this first task, a functional localizer session started during which participants were asked to perform a Monetary Incentive Delay task (MID^27^) based on a 2 (Valence: Gain vs. Loss) × 2 (Magnitude: High vs. Low) factorial structure. Specific brain targets for this MID functional localizer were the ventral striatum and the caudate (nucleus/body/head). Sixteen repetitions of each of the 4 types of trials were presented in a fully randomized order. Participants first read the instructions and completed a practice session of 8 trials before beginning the experimental session. During each trial, participants first viewed a fixation cross which was followed by a 2-s incentive valence (i.e., gain or loss) with different magnitude (+∕−0.1 CHF, +∕−2 CHF). This part of the trial was followed by a fixation cross (2 to 2.5 s; “anticipation phase”). After this part, a star was rapidly displayed on the screen (the duration of presentation of this star was initially set to the averaged time response observed in the practice session). If the participants pressed the button before the target offset, they either earned or avoided losing the amount of money previously displayed on the screen. After the initial trial, the duration of the presentation of the star was adapted in the following way: when participants managed to answer on time for three consecutive trials, the presentation of the star for the following trial was reduced by 30 ms. If participants were not fast enough, the duration of the star presentation was increased by 30 ms. Feedback indicating the trial outcome was then presented (2 s; “outcome” phase). Trials were separated from one another by an inter-trial interval ranging from 2 to 6 s. Hit rate was targeted to 66% for each participant by a function that adaptively changed target durations, depending on the performance of the participant within each condition.

### Experimental procedure: Forced-choice task (outside the MRI scanner)

Once outside the scanner, participants went through a binary choice paradigm (forced-choice task) on a computer in a cubicle. In this task, two items with their brands were presented side by side and participants were asked to select the item they would like to get. No information was given about the price of the items. Participants could take as much time as they wanted to make their choices. Each trial consisted in the presentation of one item with a luxurious brand and another item with a non-luxurious brand, with each item displayed with the same brand as during the liking task. The pairing of the items was pseudo-randomized across participants. The side of appearance on the screen of the luxurious vs non-luxurious condition was randomized across participants. Finally, participants completed a brand knowledge evaluation task where each brand logo was presented on the screen and participants were asked to assess whether they knew the brand (“1”) or not (“0”). The total mean proportion of knowledge between participants was computed and included in a regression analysis with materialism as covariate, revealing a weak tendency (b = 0.023, t(35) = 1.741, p = 0.090). This ensured that globally all participants knew well the brands used in our experiment.

### Behavioural data analysis

R^50^, lmerTest^51^, and lme4^52^ packages were used to perform a generalized linear mixed model on the liking variable (liking task). Brand label (luxurious vs. non-luxurious) was introduced as a fixed effect factor, Materialistic tendencies as covariate. Participants and stimuli were introduced as random effects. Moreover, as advised by Barr^53^ and Winter^54^, we introduced a random slope for the brand for each participant, as this allows the effect of brand to differ between participants. Participants and stimuli were introduced as random effects. A second model was computed on the choice variable (offline choice task), where the dichotomic dependent variable referred to the choice of the item displayed on the left for each trial (i.e. chosen “1”, non-chosen “0” as in Krajbich & Rangel^55^). Participants chose slightly more often products displayed on the right, thus making the proportion of luxurious and non-luxurious chosen items slightly different from one (see Fig. 1). In our model, brand (luxurious vs. non-luxurious) and liking (most vs. least liked) were introduced as factors and materialism as a covariate. Intercepts for the participants as well as for the left pictures of each choice were introduced as random intercepts. We did not include random slopes in this model due to convergence issues. In all models, we assigned the coding −1/+ 1 for fixed effects as advised by Judd *et al*.^56^, which allowed us to interpret the effects as main effects. Finally, we initially included global brand familiarity as a controlling variable in both models. However, as this variable showed no significant impact (b = −110.038, t(36.480) = −1.124, p = 0.264 for the liking variable and b = −0.388, z = −1.491, p = 0.136 for the probability of choice) and no significant improvement of the model (Delta χ 2 = 1.2129, p = 0.271, and Delta χ 2 = 2.2314, p = 0.135, respectively), it was later removed from both models.

### Image acquisition

Structural and functional brain imaging data were acquired in a 3 T scanner (Siemens Trio, Erlangen, Germany) with a 32-channel coil. A magnetization prepared rapid acquisition gradient echo sequence was used to acquire high-resolution (1 × 1 × 1 mm^3^) T1-weighted structural images (TR = 1,900 ms, TE = 2.27 ms, TI = 900 ms). Functional images were acquired with a multislice echo planar imaging sequence (36 transversal slices in descending order, slice thickness 2 mm, TR = 2,100 ms, TE = 30 ms, field of view = 205 × 205 mm^2^, 64 × 64 matrix, flip angle = 90°, bandwidth 1562 Hz/Px).

### Image analysis

#### Liking task

Functional images were analyzed with Statistical Parametric Mapping software (SPM12, Wellcome Trust Centre for Neuroimaging, London, UK, http://www.fil.ion.ucl.ac.uk/spm/). Preprocessing steps included realignment to the first volume of the time series, slice timing, normalization to the Montreal Neurological Institute (MNI)^57^ space using the DARTEL toolbox^58^ and spatial smoothing with an isotropic Gaussian filter of 8 mm full width at half maximum. To remove low frequency components, we used a high-pass filter with a cutoff frequency of 128 s. Anatomical locations were defined with a standardized coordinate database (Talairach Client, http://www.talairach.org/client.html) transforming MNI coordinates to the Talairach space and transforming it back into MNI for display purposes. For this fMRI liking task, we used a first-level general linear model, in which each stimulus display was modeled by using a stick function and was convolved with the hemodynamic response function. Events were time-locked to the onset of the display of stimuli (duration 4 seconds) because it most likely reflects the automatic evaluation process^21^ and the evaluation phase was included in the model as well. Separate regressors were created for each experimental condition (i.e., luxurious vs. non-luxurious brand label) and were merged with regressors assessing the Liking of each participant for each trial (i.e., most liked vs. least liked). We performed a median split for each participant’s evaluations for both luxurious/non-luxurious items, and further tagged the evaluations above this median as “most liked” while evaluations lower than the median were tagged as “least liked”. We thus had four regressors of interest (luxurious most liked, luxurious least liked, non-luxurious most liked, non-luxurious least liked) including 20 trials each and 80 trials in total in addition to an identical number of conditions with the onset locked to the Liking phase, as non-interest regressors. Moreover, six motion parameters were included as regressors of no interest to account for movement in the data. The neuroimaging data were analyzed using three different models in order to accurately characterize neural networks of subjective value and to address our hypotheses as accurately and reliably as possible.

In a first model, the four regressors of interest were used to compute linear simple contrasts for each participant and were then taken to a second-level, flexible factorial analysis. The second-level analysis was performed with a 2 × 2 factorial design with the factors “Liking” (most vs. least liked) and “Brand label” (luxurious vs. non-luxurious). We computed the interaction between luxury and Liking factors because we were specifically interested in the contrasts [most liked > least liked * luxurious > non-luxurious; least liked > most liked * luxurious > non-luxurious]. The impact of materialism was not tested in this model, as it was strictly designed to answer to our first hypothesis (i.e. how brands randomly displayed with items may have an impact on the brain valuation system).

In a second model, a functional localizer designed to localize reward-related brain areas (MID task^27^) was used to define group-level regions of interest (ROIs) used for each participant, based on significantly higher activations for High than Low reward, thresholded to *p < *0.05, with voxel-wise family wise error (FWE) correction, and an arbitrary cluster extent of k > 10 voxels. ROIs were defined in the left and right caudate nucleus, the global maxima of High reward > Low reward. These ROIs were defined independently of the liking task, and were hence specific to our sample. Because the ROIs were extracted for data of model 1, the number of trials (N_t_) was balanced for the four regressors (Most liked luxurious: N_t_ = 20; Least liked luxurious: N_t_ = 20; Most liked non-luxurious: N_t_ = 20; Least liked non-luxurious: N_t_ = 20). A posteriori percentage of signal change analysis (for each peak separately and each condition) was performed by using repeated-measure ANOVAs, and post hoc correction for multiple comparisons was applied by using a Tukey HSD correction following normality estimation.

In a third model, we took into account information related to the choices performed outside the scanner. This model was based on the following regressors of interest: “Choice” (chosen; not-chosen), “Brand label” (luxurious; non-luxurious). The first-level design matrix included hence 4 columns (luxurious chosen, mean number of trials = 21, range 16–26; luxurious not-chosen, mean number of trials = 19, range 14–24; non-luxurious chosen, mean number of trials = 21, range 14–24; non-luxurious not-chosen, mean number of trials = 19, range 16–26). The 2-way interaction between these regressors was computed at the first-level of analysis for all participants. Then, “Materialism” was used as a second-level covariate in the model (second-level analysis) to test our second hypothesis. “Materialism” was set to interact with Factor 1 in this second-level analysis, namely the factor including the 2-way interaction between “Choice” and “Brand label” that was computed at the first-level. By doing so, we were able to display enhanced brain activity for Choice * Brand label ([chosen > non-chosen * luxurious > non-luxurious]), which was further scaled by Materialism hence leading to the 3-way interaction we were aiming for.

For all three models, the second-level flexible factorial design assumed that “participants” (Factor 1) were independent whereas “conditions” (Factor 2) were not. Variance estimation was set to unequal for all factors in order to take into account the inhomogeneous variance of the data. The reported neuroimaging activations of model 1 was threshold in SPM12 using voxel-wise false discovery rate (FDR) correction at *p* < 0.05 to account for multiple comparisons. To remove single voxels or very small clusters and hence reduce even more the 5% risk of false positives among significant voxels specific to FDR, an arbitrary cluster extent of k > 50 voxels was used. For model 2, a ROI analysis was performed as mentioned above and results were submitted to a repeated-measure ANOVA based on extracted signal in the bilateral caudate head. Reported statistics show HSD Tukey-corrected values of p < 0.001 for all significant interactions. For model 3, we displayed the results at an uncorrected threshold at *p* < 0.001 and then performed a small volume correction (Psvc < 0.05 FDR at the voxel level) using an anatomical image of the bilateral striatum, ventral and dorsal portions, and the VMPFC computed using the anatomy toolbox^59^, more specifically the IBASPM atlas.

#### Monetary Incentive Delay task

Preprocessing steps were in every aspect identical to those of the liking task. Anatomical locations were defined with a standardized coordinate database (Talairach Client, http://www.talairach.org/client.html) transforming MNI coordinates to the Talairach space and transforming it back into MNI for display purposes. We used a first-level general linear model, in which each event was modeled by using a stick function and was convolved with the hemodynamic response function. Events were time-locked to the onset of the display of the cue, corresponding to the anticipation of reward^60^. Separate regressors were created for each experimental condition modalities (High vs low reward, Gain vs loss). The four regressors of interest were used to compute linear simple contrasts for each participant and were then taken to a second-level, flexible factorial analysis. Six motion parameters were included as regressors of no interest to account for movement in the data. The second-level analysis was performed with a 2 × 2 factorial design with the factors “Reward” (high vs. low) and “Outcome” (gain vs. loss). We computed the interaction between “Reward” and “Outcome” factors but we were specifically interested in the first factor hence in the contrast [High > Low reward]. The second-level flexible factorial design assumed that “participants” (Factor 1) were independent whereas “Reward” (Factor 2) and “Outcome” (Factor 3) were not. Variance estimation was set to unequal for all factors in order to take into account the inhomogeneous variance of the data. Reported neuroimaging activations were threshold in SPM12 using Family-Wise Error (FWE) correction at p < 0.05 at the voxel level to account for multiple comparisons. To remove single voxels or very small clusters, an arbitrary cluster extent of k > 10 voxels was used.

#### Percentage of signal change extraction for liking and MID tasks

For both tasks and all reported contrasts, percentage of signal change was extracted for each condition by first isolating the local maximum of interest. The MNI *xyz* coordinates of the peak voxel were selected and 9 contiguous voxels explaining at least 95% of the variance were included using the singular value decomposition method^61^. Percentage of signal change was then averaged for each condition and for all participants. The grand average is used and displayed in the figures including the standard error of the mean as indicated by error bars, except for Fig. 4 in which one mean value per participant and per condition is displayed (4 values per participant) to render second-level analyses including Materialism as covariate.

### Data availability statement

Behavioral data and analysis codes (R codes for behavior and Matlab and SPM12 batches and scripts for fMRI data) are available online as supplementary information. MRI data are available from the corresponding author upon reasonable request.

## References

[1] Bredahl L (2004). Cue utilisation and quality perception with regard to branded beef. Food Qual. Prefer..

[2] Zeithaml VA (1988). Consumer perceptions of price, quality, and value: a means-end model and synthesis of evidence. J. Mark..

[3] Plassmann H, O’Doherty J, Shiv B, Rangel A (2008). Marketing actions can modulate neural representations of experienced pleasantness. Proc. Natl. Acad. Sci..

[4] Lee N, Broderick AJ, Chamberlain L (2007). What is [] neuromarketing’? A discussion and agenda for future research. Int. J. Psychophysiol..

[5] Linder NS (2010). Organic labeling influences food valuation and choice. NeuroImage.

[6] Sörqvist P (2015). The green halo: Mechanisms and limits of the eco-label effect. Food Qual. Prefer..

[7] Sörqvist P, Haga A, Holmgren M, Hansla A (2015). An eco-label effect in the built environment: Performance and comfort effects of labeling a light source environmentally friendly. J. Environ. Psychol..

[8] Sörqvist, P. *et al*. Who needs cream and sugar when there is eco-labeling? *Taste and willingness to pay for ‘eco-friendly’ coffee*. **8** (2013). 10.1371/journal.pone.0080719 PMC3851458 24324623

[9] Allison RI, Uhl KP (1964). Influence of beer brand identification on taste perception. J. Mark. Res..

[10] McClure SM (2004). Neural correlates of behavioral preference for culturally familiar drinks. Neuron.

[11] Kuhnen CM, Knutson B (2005). The neural basis of financial risk taking. Neuron.

[12] Bartra O, McGuire JT, Kable JW (2013). The valuation system: a coordinate-based meta-analysis of BOLD fMRI experiments examining neural correlates of subjective value. Neuroimage.

[13] Brosch T, Sander D (2013). Neurocognitive mechanisms underlying value-based decision-making: from core values to economic value. Front. Hum. Neurosci..

[14] Kable JW, Glimcher PW (2007). The neural correlates of subjective value during intertemporal choice. Nat. Neurosci..

[15] Levy DJ, Glimcher PW (2012). The root of all value: a neural common currency for choice. Curr. Opin. Neurobiol..

[16] Sescousse G, Caldú X, Segura B, Dreher J-C (2013). Processing of primary and secondary rewards: a quantitative meta-analysis and review of human functional neuroimaging studies. Neurosci. Biobehav. Rev..

[17] Sescousse G, Redouté J, Dreher J-C (2010). The architecture of reward value coding in the human orbitofrontal cortex. J. Neurosci..

[18] Plassmann H, O’Doherty JP, Rangel A (2010). Appetitive and aversive goal values are encoded in the medial orbitofrontal cortex at the time of decision making. J. Neurosci..

[19] Chib VS, Rangel A, Shimojo S, O’Doherty JP (2009). Evidence for a common representation of decision values for dissimilar goods in human ventromedial prefrontal cortex. J. Neurosci..

[20] Erk S, Spitzer M, Wunderlich AP, Galley L, Walter H (2002). Cultural objects modulate reward circuitry. Neuroreport.

[21] Lebreton M, Jorge S, Michel V, Thirion B, Pessiglione M (2009). An automatic valuation system in the human brain: evidence from functional neuroimaging. Neuron.

[22] Izuma K, Saito DN, Sadato N (2008). Processing of social and monetary rewards in the human striatum. Neuron.

[23] Kirk U, Harvey A (2011). & Montague, P. R. Domain expertise insulates against judgment bias by monetary favors through a modulation of ventromedial prefrontal cortex. Proc. Natl. Acad. Sci..

[24] Rindfleisch A, Burroughs JE, Wong N (2009). The safety of objects: Materialism, existential insecurity, and brand connection. J. Consum. Res..

[25] Ostrovskaya, L. & Sarabia, F. *Effect of materialism on the use of the brand name in purchasing decisions from a cross-cultural perspective*. (2013).

[26] Audrin C, Brosch T, Chanal J, Sander D (2017). When symbolism overtakes quality: Materialists consumers disregard product quality when faced with luxury brands. J. Econ. Psychol..

[27] Knutson B, Westdorp A, Kaiser E, Hommer D (2000). FMRI visualization of brain activity during a monetary incentive delay task. Neuroimage.

[28] Hahnel UJ, Gölz S, Spada H (2014). How does green suit me? Consumers mentally match perceived product attributes with their domain-specific motives when making green purchase decisions. J. Consum. Behav..

[29] Fink GR (1996). Where in the brain does visual attention select the forest and the trees?. Nature.

[30] Rudorf S, Hare TA (2014). Interactions between Dorsolateral and Ventromedial Prefrontal Cortex Underlie Context-Dependent Stimulus Valuation in Goal-Directed Choice. J. Neurosci..

[31] Clithero, J. A. & Rangel, A. Informatic parcellation of the network involved in the computation of subjective value. *Soc. Cogn. Affect. Neurosci.* **9**, 1289–1302 (2014). 10.1093/scan/nst106 PMC4158359 23887811

[32] Schultz W (2000). Multiple reward signals in the brain. Nat. Rev. Neurosci..

[33] Delgado MR, Locke HM, Stenger VA, Fiez JA (2003). Dorsal striatum responses to reward and punishment: effects of valence and magnitude manipulations. Cogn. Affect. Behav. Neurosci..

[34] Gottfried JA, O’Doherty J, Dolan RJ (2003). Encoding predictive reward value in human amygdala and orbitofrontal cortex. Science.

[35] Schmidt, L., Skvortsova, V., Kullen, C., Weber, B. & Plassmann, H. How context alters value: Price information recruits the brain’s valuation and affective regulation system for shaping experienced taste pleasantness. *bioRxiv* 9791510.1101/097915 (2017). 10.1038/s41598-017-08080-0 PMC5556089 28808246

[36] Kirk, U. & Freedberg, D. A. Contextual bias and insulation against bias during aesthetic rating. The roles of VMPFC and DLPFC in neural valuation. *Art Aesthet*. *Brain* 158–173, 10.1093/acprof:oso/9780199670000.003.0008 (2015).

[37] Roy M, Shohamy D, Wager TD (2012). Ventromedial prefrontal-subcortical systems and the generation of affective meaning. Trends Cogn. Sci..

[38] Lebreton M (2013). A Critical Role for the Hippocampus in the Valuation of Imagined Outcomes. PLOS Biol..

[39] Palombo DJ, Keane MM, Verfaellie M (2015). How does the hippocampus shape decisions?. Neurobiol. Learn. Mem..

[40] Vartanian O, Goel V (2004). Neuroanatomical correlates of aesthetic preference for paintings. Neuroreport.

[41] Park B, Tsai JL, Chim L, Blevins E, Knutson B (2016). Neural evidence for cultural differences in the valuation of positive facial expressions. Soc. Cogn. Affect. Neurosci..

[42] Freeman JB, Rule NO, Adams RB, Ambady N (2009). Culture shapes a mesolimbic response to signals of dominance and subordination that associates with behavior. NeuroImage.

[43] Gil LA, Kwon K-N, Good LK, Johnson LW (2012). Impact of self on attitudes toward luxury brands among teens. J. Bus. Res..

[44] Brosch T, Coppin G, Scherer KR, Schwartz S, Sander D (2011). Generating value (s): Psychological value hierarchies reflect context-dependent sensitivity of the reward system. Soc. Neurosci..

[45] Sharot T, Martino BD, Dolan RJ (2009). How Choice Reveals and Shapes Expected Hedonic Outcome. J. Neurosci..

[46] Kasser T, Ryan RM (1993). A dark side of the American dream: correlates of financial success as a central life aspiration. J. Pers. Soc. Psychol..

[47] Grouzet FME (2005). The Structure of Goal Contents Across 15 Cultures. J. Pers. Soc. Psychol..

[48] Kasser T, Ahuvia A (2002). Materialistic values and well-being in business students. Eur. J. Soc. Psychol..

[49] L2 Think Tank, & NYU Stren. *Gen Y Prestige Brand Ranking 2010*. (2010).

[50] R Development Core Team. *R: A language and environment for statistical computing*. (R Foundation for Statistical Computing, 2008).

[51] Kuznetsova, A., Brockhoff, P. B. & Christensen, R. H. B. *lmerTest: Tests for random and fixed effects for linear mixed effect models* (*lmer objects of lme4 package*). *R package version 1*.*0-2* (2013).

[52] Bates, D., Maechler, M., Bolker, B. & Walker, S. lme4: Linear mixed-effects models using Eigen and S4. *J*. *Stat*. *Softw*. **67** (2015).

[53] Barr, D. J. Random effects structure for testing interactions in linear mixed-effects models. *Front*. *Psychol*. **4** (2013). 10.3389/fpsyg.2013.00328 PMC3672519 23761778

[54] Winter, B. *Linear models and linear mixed effects models in R with linguistic applications*. (2013).

[55] Krajbich I, Rangel A (2011). Multialternative drift-diffusion model predicts the relationship between visual fixations and choice in value-based decisions. Proc. Natl. Acad. Sci..

[56] Judd CM, Westfall J, Kenny DA (2012). Treating stimuli as a random factor in social psychology: a new and comprehensive solution to a pervasive but largely ignored problem. J. Pers. Soc. Psychol..

[57] Collins DL, Neelin P, Peters TM, Evans AC (1994). Automatic 3D intersubject registration of MR volumetric data in standardized Talairach space. J. Comput. Assist. Tomogr..

[58] Ashburner J (2007). A fast diffeomorphic image registration algorithm. Neuroimage.

[59] Eickhoff SB (2005). A new SPM toolbox for combining probabilistic cytoarchitectonic maps and functional imaging data. NeuroImage.

[60] Knutson B, Fong GW, Adams CM, Varner JL, Hommer D (2001). Dissociation of reward anticipation and outcome with event-related fMRI. NeuroReport.

[61] Golub GH, Reinsch C (1970). Singular value decomposition and least squares solutions. Numer. Math..

